# The Intertemporal Role of Respiratory Support in Improving Neonatal Outcomes: A Narrative Review

**DOI:** 10.3390/children8100883

**Published:** 2021-10-02

**Authors:** Kosmas Sarafidis, William Chotas, Eleni Agakidou, Paraskevi Karagianni, Vasiliki Drossou

**Affiliations:** 1 1st Department of Neonatology and Neonatal Intensive Care, School of Medicine, Aristotle University of Thessaloniki, Ippokrateion General Hospital, 54642 Thessaloniki, Greece; eagaki@hotmail.com (E.A.); karagpar@gmail.com (P.K.); agakidou@gmail.com (V.D.); 2 Department of Neonatology, University of Vermont, Burlington, VT 05405, USA; wchotas@gmail.com

**Keywords:** bronchopulmonary dysplasia, mechanical ventilation, neonate, non-invasive ventilation, prematurity, outcome, respiratory distress

## Abstract

Defining improvements in healthcare can be challenging due to the need to assess multiple outcomes and measures. In neonates, although progress in respiratory support has been a key factor in improving survival, the same degree of improvement has not been documented in certain outcomes, such as bronchopulmonary dysplasia. By exploring the evolution of neonatal respiratory care over the last 60 years, this review highlights not only the scientific advances that occurred with the application of invasive mechanical ventilation but also the weakness of the existing knowledge. The contributing role of non-invasive ventilation and less-invasive surfactant administration methods as well as of certain pharmacological therapies is also discussed. Moreover, we analyze the cost–benefit of neonatal care-respiratory support and present future challenges and perspectives.

## 1. Introduction

**Figure 1:**
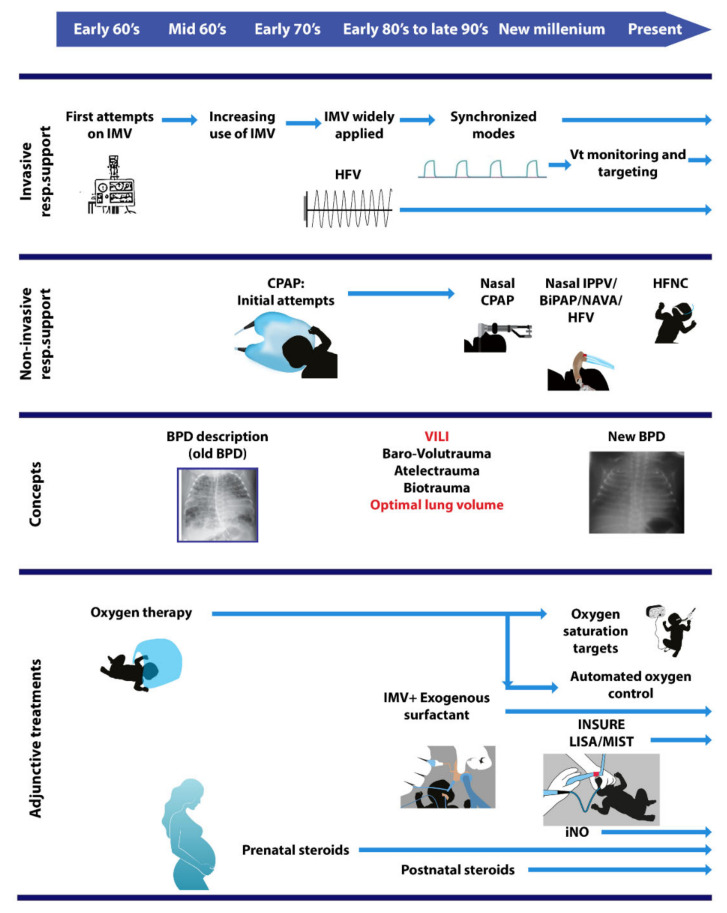
Schematic presentation of the intertemporal evolution of neonatal respiratory management from the early 1960s to the present. BiPAP, Bi-level positive airway pressure; BPD, bronchopulmonary dysplasia; CPAP, continuous positive airway pressure; HFNC, high flow nasal canula; HFV, high-frequency ventilation; IPPV, intermittent positive pressure ventilation; iNO, inhaled nitric oxide; IMV, invasive mechanical ventilation; NAVA, neurally adjusted ventilatory assist; Vt, tidal volume; VILI, ventilator-induced lung injury.

Improving patient outcomes is an issue every health care system strives for but defining “improvements” is sometimes difficult. While improvement can be simply defined as mortality reduction or as a specific favorable outcome after an intervention, it can also have a broad definition incorporating many different measures. Regarding respiratory support in neonates, the question on the improvement in neonatal outcomes is difficult to answer. Although efforts to review the effects of invasive mechanical ventilation (IMV) on neonates have been made in the past [1], the challenging, exciting, and frustrating evolution of neonatal respiratory support up to the present day (Figure 1) renders the balance of benefits and harms a moving target. In this narrative review, we describe the intertemporal role of the various modes of respiratory support having been used so far in neonates (especially the mechanical ventilation) on survival and other important clinical outcomes, highlighting the existing shortfalls and gaps of knowledge that need to be addressed in future research.

## 2. Effect of IMV on Neonatal Survival

Nowadays, it would be hard to imagine a late-premature infant born at 34 weeks gestation, with a birth weight (BW) of 2100 g, dying of respiratory distress syndrome (RDS). This was the case of the son of the United States President John Fitzgerald Kennedy. The child received hyperbaric treatment, but despite frantic efforts by physicians, he only lived for 39 h (7 August 1963–9 August 1963). A young doctor working at the Hospital for Sick Children in Toronto, Canada, Dr. Maria Delivoria-Papadopoulos (1930–2020), who, along with Paul Swyer, was the first to introduce assisted ventilation in neonates successfully, was also called. However, due to various reasons in that time era, it was not possible to get involved in the infant’s care [2,3].

In the 1960s, the role of IMV in neonatal survival was not yet known, but it seemed to be helpful in newborns with a BW greater than 2 kg [1]. At the same time, and along with the initial implementation of IMV, it became obvious that surviving newborns suffered from severe lung damage. This was described by Northway et al. in 1967 and termed “bronchopulmonary dysplasia” (BPD) [4]. Interestingly, in early studies performed in the same time period, IMV was not found to be associated with other side-effects and complications, such as intraventricular hemorrhage (IVH) [1], presumably due to the fact that head ultrasound was not yet performed, and brain injury was only seen in autopsy.

Two medical breakthroughs in the 1970s considerably affected the outcome of premature newborns: continuous positive airway pressure (CPAP) [5] and the administration of antenatal steroids [6]. Further progress was made with the invention of exogenous surfactants to treat neonatal RDS [7] and its wide introduction into clinical practice in the ensuing decades. Following the reduction in the severity of neonatal RDS, other advances in perinatal and neonatal care allowed for a dramatic increase in survival of very premature newborns in the late 1980s and early 1990s [8].

During the following decades, there was even more progress made worldwide, with improved overall survival of premature infants in developed countries [9,10]. Today, the survival of extremely premature infants has reached the point that previously seemed impossible—the limits of viability have shifted. Data from the Vermont Oxford Network (VON) (2000–2009) showed a significant decrease in infant mortality with BWs 501 to 1500 g from 14.3% to 12.4% (within each of the 250 g BW categories except in the highest BW category (1251 to 1500 g) as mortality was already low). Not surprisingly, the mortality rate was inversely related to BW [11].

Similar findings were reported by the Eunice Kennedy Shriver National Institute of Child Health and Human Development (NIHCD) for the years 1993 to 2012. In the latter study, survival increased most markedly for infants born at 23 and 24 weeks’ gestation, while survival without major morbidity increased for infants aged 25 to 28 weeks. Of note, in this cohort, most infants who survived more than 12 h were mechanically ventilated, which indicates the crucial role of IMV in improving survival [12]. Moreover, a recent retrospective study reported on the trends in epidemiology and outcomes of RDS in preterm infants with gestational age (GA) ≤ 34 weeks born in the United States between 2003 and 2014. A high proportion of infants (56.6%) received IMV even in the time period 2012 to 2014, while an increasing trend of non-invasive ventilation (NIV) was observed. It is worth noting that despite the decrease in all-cause mortality over time, the in-hospital mortality was found to be higher in neonates who received IMV [13], presumably due to more severe respiratory disease.

According to a meta-analysis involving cohort studies published between 2000 and 2017 in which data on survival or neurodevelopmental outcomes were provided, survival without impairment for infants born alive in high-income countries increased from 1.2% to 9.3% for 22 to 24 weeks’ gestation, and from 40.6% to 64.2% for 25 to 27 weeks’ gestation. Although one could assume that high rates of IMV were used in the lowest gestational ages, the latter meta-analysis did not report on the respiratory support modes applied [14]. Similarly, in the EPIPAGE-2 study, a prospective, a French population-based cohort study of infants born alive at 22 through 34 weeks’ gestation, 96.3%, 58.7% and 12.7% of the infants born, respectively, at 22–26, 27–31 and 32–34 weeks’ gestation in 2011 received exogenous surfactant [15], presumably following tracheal intubation and IMV as less invasive techniques were not widely used at that time.

Lastly, results of a recently published cohort study which evaluated trends in outcomes of very preterm and very low birth weight (VLBW) neonates born between 2007–2011 and 2012–2015 in 11 high-income countries showed a significant decrease in mortality during the last epoch in most, but not all countries. As no additional data regarding respiratory support modes are provided in the study, the contributing role of IMV cannot be clarified [16]. 

Irrespective of the changes that occurred over time in maternal and neonatal care, IMV has positively affected neonatal mortality. 

## 3. Effect of Intertemporal Changes in IMV Associated Morbidities

Several complications have been described in all age groups due to the application of IMV. In neonates, especially preterm ones, BPD and brain injury are the morbidities of most interest in regard to IMV due to the pathophysiological association with IMV and their significant clinical consequences.

### 3.1. Bronchopulmonary Dysplasia

Before focusing on the intertemporal trends in BPD, one should also note the change in the disease phenotype. In 1999, Alan Jobe—in contrast to the “old BPD” reported by Northway et al.—used the term “new BPD” to describe a progressive lung injury syndrome seen in extremely preterm infants that was characterized by an arrest in lung development and minimal fibrosis [17].

Moreover, given that neonatal care practices and potential outcomes have changed over the last 20 to 25 years, data related to the impact of IMV on BPD should be time-period specific. Previous reports from the VON database did show a slight decrease in BPD by 1.4% between 2000 and 2009; however, the incidence of BPD remained high (26.3%) in newborns weighing 500–1500 g [11]. Similarly, Stoll et al. reported that among eight centers in the Neonatal Research Network, the diagnosis of BPD rose from 32% in 1993 to 45% in 2000 but decreased to 40% in 2008. Between 2009 and 2012, BPD rates increased significantly for infants born at 26 weeks’ gestation from 50% to 55% and for those born at 27 weeks from 33% to 40%. However, there was no increase seen in infants born at 22 to 25 weeks or 28 weeks [12].

Other recently published data from the United States indicated a significant decrease in the incidence of BPD (from 14% in 2003 to 12.5% in 2014) in preterm infants ≤ 34 weeks’ gestation with RDS. Still, the trend in BPD was not that impressive in preterm infants < 28 weeks GA, as the incidence of BPD in this age group, remained the same (32.8%) for the 2009–2011 and 2012–2014 time periods [13]. A modest but significant decrease in BPD from 17.3% to 16% between 2001 and 2016 was recently documented by NEOCOSUR centers, a South American neonatal network, as well. Additionally, in the latter study, although the overall mortality of VLBW infants remained unchanged over a 16-year period, a significant improvement in survival without major morbidity was observed [18]. National data from France from 2011 showed the development of severe BPD in 25.6% of the survivors born at 24–26 week’s gestation [15]. Another study involving high-income countries reported an increased rate of BPD over time in the majority of participating centers when the 2007–2011 and 2012–2015 epochs were compared [16].

In a recent systematic review, the global incidence of BPD was found to range between 10 and 89%. In Europe it ranged from 10 to 73%; in North America, 18 to 89%; in Asia, 18 to 82%; and in Oceania, 30 to 62%. The wide variation reported globally most possibly reflects differences in the GA and BW of infants across study populations, as well as different diagnostic criteria and care practices between study institutions [19].

In a very recent retrospective study involving 17,952 infants (GA 23 to 31 weeks) born in Spain between 2010 and 2019, no significant change in moderate/severe BPD in surviving neonates was observed over time [20].

The effect of IMV on the incidence of BPD compared to NIV has been documented by RCTs and is discussed in Section 4 on non-invasive respiratory support. Overall, despite the improvement in neonatal care, the burden of BPD remains high, partly reflecting the increased survival of extremely preterm infants. This fact not only indicates the ongoing need for further advancements of preventive and therapeutic measures but also the necessity for reliable and early diagnostic and prognostic biomarkers of the disease.

### 3.2. Brain Injury

Beyond lung damage, prolonged IMV has been associated with impaired neurodevelopment outcomes. The nature of ventilation-induced brain injury is not fully understood, as it is difficult to determine whether brain injury may be attributed to ventilation or other confounding factors of neonatal care.

Ventilation-induced brain injury in neonates involves a complex inflammatory cascade and hemodynamic instability [21]. In a retrospective cohort study of preterm infants born between 2010 and 2015 at 24 to 30 weeks gestation who received restrictive IMV (median duration of 2 days), 33% of infants who were diagnosed with moderate/severe BPD demonstrated that the duration of IMV was negatively correlated with neurodevelopmental outcome at 24 months corrected age [22]. In addition, prolonged IMV in VLBW infants was associated with retinopathy of prematurity (ROP) requiring laser therapy, periventricular leukomalacia, abnormal auditory screening tests, increased length of hospitalization, parenteral nutrition, and a higher probability of discharge with poor achievement of physical growth [23]. 

On the other hand, BPD itself has been associated with an increased risk of neurodevelopmental impairment. Although it is difficult to demonstrate a direct effect of IMV on the complications of prematurity, in preterm infants born at less than 29 weeks gestation, the absence of three major neonatal morbidities—BPD, necrotizing enterocolitis (NEC), and severe neurological injury—has been associated with a good neurodevelopmental outcome at 18 months corrected age. For every week reduction in positive pressure ventilatory support, there was a 2 to 10% increase in the odds of achieving a good outcome [24].

## 4. Most Important Developments in IMV and Their Effect on Outcomes

Given the direct association of IMV with organ damage, especially in the immature neonatal lung, research along with technological advances in the field of neonatal ventilators allowed for progression toward the introduction of new concepts and the implementation of new ventilatory techniques (Table 1).

### 4.1. New Concepts

Pivotal studies on lung injury in adults performed in the 1990s revolutionized respiratory management in all age groups, including neonates. These studies showed that high volume ventilation was more injurious to the lungs than high pressure [66], thus introducing the term “volume trauma or volutrauma”. Moreover, in patients with early acute (adult) respiratory distress syndrome, protective ventilation strategies that targeted a tidal volume (Vt) of less than 6 mL per kg were found to increase survival at 28 days, improve the likelihood of weaning from mechanical ventilation, and lower the risk of barotrauma [67]. Animal studies identified additional important risk factors of IMV-associated lung injury—also termed ventilator-induced lung injury (VILI)—such as atelectrauma, biotrauma attributed to IMV—induced release of mediators, which cause or worsen lung injury and oxygen toxicity. Enhanced by surfactant dysfunction, VILI is considered as one of the major risk factors for the development of BPD [68,69].

Based on this research and the technological progress made over time, respiratory monitoring, including Vt measurement and targeting, was gradually introduced into the field of neonatology (see “Volume-targeted ventilation” below). In a previous study evaluating ventilation practices in European neonatal intensive care units, Vt was measured in 84% of the patients on conventional ventilation [69].

Additionally, due to the growing concern regarding the association between hypocapnia (associated with high pressures and volumes) and increased BPD, but also neurodevelopmental morbidity [70,71], the practice of allowing higher pCO_2_ levels has been applied—the so-called “Permissive hypercapnia”. Interestingly, although not shown to reduce the incidence of BPD [72], this approach has been widely accepted by neonatologists in practice. A randomized controlled multicenter trial in mechanically ventilated extremely low birth weight (ELBW) infants, assigned to either a high pCO₂ target or control group, failed to document any benefit on lung protection, nor differences in mortality, IVH, or ROP between groups. Interestingly, the study was stopped during the interim analysis as significantly more infants developed NEC in the higher pCO₂ group [73]. As was shown in a follow-up study by the same group of investigators, the higher pCO_2_ target did not influence neurodevelopmental outcomes in the studied infants [74]. 

### 4.2. Modern Ventilators and New Modes of IMV

Great progress has been made during the recent decades regarding the ventilators used in the care of neonates. Modern ventilators can synchronize ventilatory inflation with the patient’s inspiration, readjust for gas leakage, accurately measure small Vt, and automatically adjust peak inspiratory pressure (PIP), breathing rate, and the fraction of inspired oxygen (FiO_2_). 

#### 4.2.1. Synchronized Modes of IMV

Synchronizing positive pressure ventilation with the newborn’s respiratory efforts may—at least theoretically—reduce the need for respiratory support and lung damage. Nevertheless, lung protective strategies used during IMV have not always been as successful as expected.

Compared to conventional ventilation, the benefit has been demonstrated for both high-frequency positive pressure ventilation (HFPPV) and triggered ventilation with regard to a reduction in air leak syndromes and a shorter duration of ventilation. However, neither HFPPV nor triggered ventilation was found to significantly reduce the incidence of BPD [25]. Gas leakage and trigger delay during flow or pressure synchronization are well-recognized disadvantages of the commonly used synchronized respiratory support methods.

Neurally Adjusted Ventilatory Assist (NAVA) has also been evaluated as a mode to synchronize the neonate’s respirations with the ventilator [26]. With NAVA, an oral or nasogastric catheter is inserted, which measures the electrical activity of the diaphragm. This facilitates synchronization between the ventilator and the infant’s respiratory efforts. As NAVA allows the infant to initiate support of inspiration and terminate inspiration, potentially lower pressures are applied. Moreover, hypocarbia and hypercarbia may be avoided as the patient controls his/her own respiratory drive. However, a recent Cochrane meta-analysis, which included only one randomized controlled trial (RCT), comparing NAVA to other forms of triggered ventilation for neonatal respiratory support, reported no significant difference in the duration of mechanical ventilation, rates of BPD, pneumothorax, or IVH were [27]. Investigators of the RCT reported lower PIP delivered by NAVA compared with assist control ventilation [28].

Proportional assist ventilation (PAV) synchronizes pressure support with the patient’s respiratory demand throughout the respiratory cycle. In a recent study, both PAV and NAVA were found to improve oxygenation when compared to conventional ventilation [29]. Still, important questions remain regarding the optimal level of support required for different neonatal respiratory problems. Additionally, although NAVA can effectively alleviate the work of respiration and discomfort, the neural feedback required to prevent lung overinflation was found to be insufficient in preterm infants [75].

#### 4.2.2. Volume-Targeted Ventilation

In volume targeted ventilation (VTV), the primary target is not the positive inspiratory pressure but delivered Vt. Basically, there are two modes of VTV: volume-controlled ventilation (VCV) and volume guarantee (VG) ventilation [68]. Although VCV has been the preferable ventilation mode for older children and adults, existing data on neonates are sparse. In a past study involving 50 preterm infants with RDS weighing 1200 g or more, where VCV was tested against Pressure Limited Ventilation (PLT) with the aim of delivering a Vt of 5–8 mL/kg in both groups, infants randomized to VCV met weaning criteria sooner, had a shorter duration of IMV, and a significantly lower incidence of brain injury [76].

VG is a promising mode of mechanical ventilation that has relatively recently gained attention in neonatology. In this model, PIP is automatically adjusted by the ventilator while maintaining a constant Vt. This helps to avoid hyperventilation and hypoventilation of the lungs, reducing the risk of both atelectrauma and volutrauma.

Data on the appropriate VT for different neonatal respiratory conditions are limited. Previous studies on mechanical ventilation strategies in neonates showed that targeted Vt usually ranges between 4 to 7 mL/kg [69]. However, a survey of neonatologists practicing in the United States and Canada on VTV showed a wide range of responses when they were presented with various hypothetical scenarios and asked to choose the most appropriate initial Vt [77]. For infants with RDS managed with VG, initial Vt is recommended to be set at 5.5–6 mL/kg and 4.0–4.5 mL/kg in infants with BW < 700 g and for the larger ones, respectively [78]. Subsequent adjustments are made, usually within the range of 4.0 to 6.0 mL/kg, to achieve acceptable PaCO_2_ values [79]. However, there is evidence for higher Vt requirements with advancing postnatal age in ELBW infants as well as in infants with established severe BPD. The increase in Vt requirement is greatest during the third week of life, which is presumed to be due to enlargement of the upper airways (acquired tracheomegaly) and an increase in alveolar dead space [80]. Interestingly, in infants with evolving or established BPD who remain ventilator-dependent at or beyond seven days of age, a Vt target of 7 mL/kg was found to reduce the work of breathing compared to smaller Vt (4–6 mL/kg) [81]. The application of VG was reported to be feasible even in extremely premature infants [82,83]. Of note, according to a survey on ventilation practices, VTV was more commonly used in Canada (81%) compared with the United States (39%). A lack of knowledge on the use of VTV and a lack of appropriate equipment were reported to be the chief barriers to its use [77].

Several meta-analyses documented that the use of VTV, when compared to conventional respiratory support, significantly reduces BPD, duration of IMV, pneumothorax, the combined outcome of death/BPD, as well as severe brain damage (IVH, periventricular leukomalacia) [30,31]. Moreover, according to a systematic review and network meta-analysis of 2832 patients who received one of 16 ventilation modes, the SIMV + VG mode –followed by the VCV mode—was associated with the greatest potential to reduce the mortality of infants with RDS [32]. Additional studies need to assess whether the use of VTV improves neurodevelopmental outcomes and to compare and improve VTV techniques. 

#### 4.2.3. High-Frequency Ventilation

Three types of high-frequency ventilation (HFV) have been described with significant differences in their principles of operation: high-frequency oscillatory ventilation (HFOV), high-frequency jet ventilation (HFJV), and high-frequency flow interrupter. Still, the common feature of all types is the delivery of Vt similar to or smaller than the anatomical dead space [84]. Theoretically, this characteristic rendered HFV as a lung-protective strategy with great potential. Nevertheless, the initial enthusiasm of the scientific community in the early 1990s regarding HFOV as the primary method of treating RDS was not confirmed in subsequent studies, the majority of which showed little effect in reducing lung damage in premature infants and no advantage over the conventional IMV strategies [33]. However, a RCT by Zivanovic et al. showed that ex preterm children who had undergone HFOV had superior lung function at 11 to 14 years of age, as compared with those who had received conventional IMV [34].

An important lesson, though, that neonatologists learned from HFV (possibly leading to less “PEEP-phobia” and better application of IMV) was that the “high lung volume strategy” may improve outcomes [85], a fact that strongly indicated the significance of optimal lung recruitment. Moreover, studies showed that—in the context of lung-protective maneuvers—lungs should not only be recruited but also kept open by applying appropriate PEEP [86]. Examining alveolar mechanics, Carney et al., in a review article on dynamic alveolar mechanics and VILI, reported that his group of investigators was able to visualize unstable alveoli in animal models of lung injury directly; a dramatic improvement in alveolar stability using HFOV as compared with IMV was demonstrated [35]. The authors concluded that ventilator maneuvers that promote alveolar stability, including the application of PEEP, may reduce VILI, especially if used early in the course of the disease [35]. Thus, it is not surprising that with the optimization of the IMV strategies, HFOV appears to have reduced its comparative advantage in regard to pulmonary outcomes in preterm infants [87].

Irrespective of the effectiveness of HFOV when used electively in preterm infants with pulmonary dysfunction [33], HFOV is also being applied as a rescue therapy [88] despite the lack of sufficient data [89]. A prospective registry of a large number of extremely preterm infants in the US showed that from 2008 to 2012, some form of HFV was used in 38% of the infants who survived more than 12 h after birth [12]. According to a 2-point cross-sectional study conducted in Europe around the same period (between April 2007 and May 2008), HFV was used in 15% of preterm and term ventilated infants [69]. 

HFOV has also been investigated in conjunction with VG; clinicians set a predetermined Vt, which was then constantly maintained after automatic adjustments of the oscillation amplitude by the ventilator [90,91]. Thus far, clinical studies on the use of HFV-VG in preterm infants have focused on the optimal settings to improve the stability of Vt and pCO_2_. A single-center RCT including 20 preterm infants ventilated with either HFOV-VG or HFOV alone showed that Vt was maintained very close to the target for longer periods in the HFV-VG ventilated infants [92]. More recently, a single-center study including 17 preterm infants showed similar results [93]. The better control of Vt and pCO2 attained by the HFV-VG might protect the lung and cerebral circulation. However, thus far, no data on the effect of HFV-VG on short-and long-term outcomes are available.

## 5. Benefits from Moving toward Non-Invasive Respiratory Support

For a long time (1980 to early 2000), IMV was predominately used for the treatment of RDS. Over the past decades, however, NIV has been re-introduced to avoid intubation and IMV during the early stages of RDS, mainly in extremely preterm infants [94]. A study on epidemiology and outcomes of RDS reported an increasing trend for NIV between the years 2003 and 2014 [13]. Currently, several NIV modes are used as established therapies or are under investigation for potential benefits, as described below (Table 1).

### 5.1. Non-Invasive Modes of Mechanical Ventilation

#### 5.1.1. Nasal CPAP

Initial studies directly comparing IMV to nasal CPAP (nCPAP) showed no significant differences in the short-term complications of prematurity [95,96,97]. Meta-analyses which followed, however, proved that NIV may actually cause a small but significant reduction in BPD [38,39], confirming previous clinical observations of a lower incidence of BPD in centers with increased nCPAP use [98]. The use of nCPAP is thought to help establish and maintain functional residual capacity, improve work on breathing, and enhance gas exchange [99]. Today, there is a general trend toward the application of nCPAP instead of IMV. As documented in a Cochrane meta-analysis, when CPAP was compared to assisted ventilation both with or without exogenous surfactant, CPAP reduced BPD at 36 weeks, death or BPD, the need for mechanical ventilation, and the use of surfactant [100].

#### 5.1.2. Bi-Level Non-Invasive Ventilation

Bi-level non-invasive ventilation is a NIV strategy that includes two different modalities: nasal intermittent positive pressure ventilation (nIPPV) and bi-level positive airway pressure (BiPAP). Both have been investigated for their potential to augment the benefits of nCPAP in premature infants with RDS [42].

In nIPPV, intermittent positive pressure inflations are given on top of nCPAP. This method is believed to augment CPAP by administering a “sigh” to the infant through the delivery of mandatory ventilator breaths. There are two modes of nIPPV: non-synchronized and synchronized nIPPV [101]. PIP and PEEP settings are higher than in conventional mechanical ventilation to maintain adequate ventilation and oxygenation, as pressures are not fully delivered to the infant’s lungs (due to air escaping from mouth, nose, and into intestinal tract).

nIPPV has not been associated with an increase in the risk of complications when compared to nCPAP [102]. Early nIPPV has demonstrated superiority compared to nCPAP alone to decrease the risk of respiratory failure and the need for intubation/IMV among preterm infants with RDS without, however, lowering the risk of BPD [40]. Moreover, nIPPV, when compared to nCPAP, can reduce extubation failure and the need for re-intubation [41].

BiPAP is a mode of respiratory support where an infant breathes independently of two CPAP levels that are ≤4 cm H_2_O apart [103]. In a retrospective study comparing BiPAP versus nasal synchronized intermittent positive pressure ventilation as the primary mode of treatment for RDS in preterm infants, no differences were observed regarding the duration of ventilation, failure of NIV, adverse short/long term pulmonary outcomes, or requirement for multiple doses of surfactant [104]. In another retrospective study involving preterm infants ≤ 32 weeks’ gestation, the application of BiPAP compared to nCPAP significantly reduced the necessity of intubation in the first 72 h of life. However, regarding moderate and severe BPD, there was no difference in the incidence between the two groups [105]. In a more recent randomized controlled non-inferiority trial, looking at the clinical effectiveness and safety of nCPAP compared with BiPAP, no statistically significant difference in treatment failure was found in premature infants (30^+0^ to 34^+6^ weeks) with RDS treated within 24 h after birth [106]. BiPAP compared to nCPAP was also reported to reduce early extubation failure in preterm infants less than 30 weeks’ gestation [107]. Nevertheless, the authors of a recent systematic review and meta-analysis of RCTs on Bi-level non-invasive ventilation in neonatal RDS were inconclusive in their statement as to whether BiPAP improves RDS treatment compared with nCPAP. Of note, the meta-analysis revealed no difference between BiPAP and nCPAP in extubation failure, duration of mechanical ventilation following NIV failure, BPD, or mortality. These results, however, should be cautiously interpreted given the low to moderate quality of the studies and the fact that in most of them, BiPAP and nCPAP were not compared at an equal mean airway pressure [42].

#### 5.1.3. Nasal High-Frequency Ventilation

Another promising novel ventilation method that combines the effectiveness of high-frequency mechanical ventilation with the gentle approach of NIV is nasal high-frequency ventilation (nHFV). The exact mechanism of the nHFV function is not well understood. Reported positive physiological and biological effects of nHFV include high efficiency in removing CO_2_ (mainly from upper airways’ dead-space) in comparison with other NIV modes, smaller need for synchronization, and increase in the functional residual capacity. The lack of glottis constriction, which is demonstrated with high peak pressures on nIPPV, is a distinct advantage of nHFV. Moreover, animal studies showed that the use of non-invasive HFV was associated with improved alveolarization as compared to IMV [108,109].

Ventilators providing HFOV or HFJV have been used in various case–series studies of neonates at risk for intubation and IMV [109], while relevant RCTs are relatively sparse. In a study including 68 preterm infants (GA 30–36 weeks) with RDS, subjects in the nasal HFOV (nHFOV) group had a significantly shorter duration of NIV and less need for IMV when compared to the nCPAP group. Additionally, nHFOV reduced the incidence of IVH without increasing the incidence of other complications [43]. 

A similar study in more immature neonates (28 to 34 weeks of GA) showed that the use of nHFOV, when compared to nCPAP, did not decrease the requirement for IMV in the first 72 h after birth, although the duration of NIV was significantly less in the nHFOV group [44]. In very premature infants and those diagnosed with ARDS, nHFOV has also been reported to avoid the need for re-intubation when compared with nCPAP [110]. A randomized crossover study in preterm infants born at < 30 weeks of gestation comparing nHFOV with nCPAP found that, beyond feasibility, nHFOV in the majority of cases was associated with a significant decrease in the number of desaturations and bradycardia [111]. The meta-analysis by Li et al. showed that compared to nCPAP and BiPAP, nHFOV may significantly increase the removal of carbon dioxide and, thus, reduce the need for intubation and IMV in premature infants with RDS [45]. Interestingly, although the actual transmission of oscillations to the alveoli was considered to be minimal with nHFV [108], a recently published study in preterm infants on nHFOV was able to prove a substantial transmission of oscillatory volumes into the lung, preferentially into the right, non-gravity-dependent areas [112]. Questions remain, though, regarding the most appropriate settings, ventilator type, and diseases best treated using nHFV [113].

#### 5.1.4. High-Flow Nasal Cannula 

The high-flow nasal cannula (HFNC) is an increasingly used method of respiratory support for preterm infants. With this therapy, heated and humidified air (blended with oxygen as required) is provided to infants via small binasal prongs at flows >1 L/min and up to 8 L/min [114]. It is perceived that HFNC is similarly efficacious in preterm infants for the prevention of death, chronic lung disease, and treatment failure when compared to other forms of non-invasive respiratory support [46]. Moreover, the simpler interface of HFNC, along with the ease of application when compared to CPAP, rendered this method preferable by parents and nursing staff [114].

Further studies, however, questioned the efficacy of HFNC compared to nCPAP. In an international, multicenter, randomized, noninferiority trial involving 564 preterm infants (GA ≥ 28 weeks) with early RDS who had not received surfactant treatment, HFNC resulted in a significantly higher rate of treatment failure than with nCPAP. It is worth noting that this finding led to an early stop of trial recruitment [47]. Results of more recent studies either confirmed the inferiority of HFNC to nCPAP for preventing the escalation of respiratory support in infants with RDS during the first 72 h of life [115] or showed similar safety and efficacy in extremely premature infants after extubation [116].

A meta-analysis of 21 RCTs involving 2886 preterm infants revealed that, for primary respiratory support, the rates of treatment failure were similar between HFNC and nCPAP. It was also found that for respiratory support after extubation, CPAP was associated with a lower likelihood of treatment failure than HFNC. However, the incidences of nasal trauma and pneumothoraces in the HFNC group were significantly lower than in the nCPAP group [48]. 

Studies on how to wean HFNC are currently lacking. According to a recent systematic review and meta-analysis of 13 RCTs in which different weaning strategies were studied for successful weaning of nCPAP in preterm infants, a progressive reduction in CPAP pressure was found to possibly increase the chance of success at the first weaning attempt. However, this weaning process takes more time, and the final discontinuation of nCPAP comes at a later post-menstrual age. On the other hand, stepping down from nCPAP to HFNC shortens the duration of nCPAP treatment but is associated with a longer duration of oxygen administration [117].

#### 5.1.5. Nasal Neurally Adjusted Ventilatory Assist 

NAVA can be provided non-invasively (NIV-NAVA), as well. In a randomized, 2-center trial conducted in infants with BW ≤ 1500 g and RDS who received NIV for ≤ 48 h of life, no differences were observed between the nCPAP and NIV-NAVA groups in the primary outcome (need for IMV at ≤ 72 h of life), use of surfactant, duration of NIV support, or BPD incidence and death, even though the duration of mechanical ventilation was significantly longer in the nCPAP group [49]. In another RCT involving preterm infants (28^+0^ to 36^+6^ weeks gestation) requiring nCPAP and supplemental oxygen (FiO_2_ > 0.23) for RDS before the first 48 h of postnatal age, NIV-NAVA, when compared to nCPAP, had no significant effect on oxygen requirements or the need for IMV [50]. Larger RCTs on the efficacy and clinical utility of the specific NIV technique are required in preterm infants before NIV-NAVA can be routinely used in everyday practice. 

### 5.2. Alternative Exogenous Surfactant Administration Techniques

Almost in parallel to the initial efforts to minimize exposure to tracheal intubation and IMV in preterm infants with RDS and considering that a number of them will finally need surfactant replacement, novel techniques for its delivery have been proposed for the spontaneously breathing infants [118,119]. Hence, the mode of surfactant administration evolved—mainly during the last decade—from endotracheal surfactant administration during IMV to Intubate–SURfactant–Extubate (INSURE), and most recently the Less Invasive Surfactant Administration (LISA) or Minimal Invasive Surfactant Technique (MIST) (Table 1).

#### 5.2.1. Intubate-SURfactant-Extubate

INSURE is one of the most widely used methods to deliver surfactants to preterm infants with RDS. At least, in theory, it is considered less injurious to the immature lung, as it only requires a brief exposure to IMV, with the aim of extubating to NIV shortly thereafter. Major concerns with INSURE include not only the complications of intubation [120] but that the pre-medication used prior to intubation may delay extubation once the surfactant has been administered [51]. Indeed, clinical studies have shown that successful extubation may fail in a substantial number of infants. A systematic review on early predictors for INSURE failure in preterm infants with RDS by De Bisschop et al. reported a median failure rate of 33% (9.3–52%). ELBW and severe RDS were identified as significant risk factors for INSURE failure [52]. 

#### 5.2.2. Less Invasive Surfactant Administration/Minimal Invasive Surfactant Technique 

With LISA/MIST, “classical” tracheal intubation is avoided, and the surfactant is administered in spontaneously breathing infants via a feeding tube (or specifically designed catheter) that is advanced through the vocal cords under direct laryngoscopy. The avoidance of positive pressure ventilation, more effective dispersion of the surfactant and maintenance of the function of the larynx are considered important advantages of this approach. The OPTIMIST-A trial is an ongoing multicenter, masked RCT designed to assess the effectiveness of surfactant delivery using a semi-rigid surfactant instillation catheter briefly passed into the trachea (called the “Hobart method”) in avoiding mechanical ventilation in preterm infants 25–28 weeks gestation managed on CPAP without prior intubation [121].

The administration of analgesia and sedation during LISA/MIST is a matter of debate as it may interfere with spontaneous breathing and increase the need for IMV. Practice policies of using these drugs vary widely between countries and neonatal intensive care units (NICUs) [122]. In general, LISA has been widely adopted in Europe, and, recently, considerable efforts have been made by neonatologists to incorporate this practice in the US, as well [123]. 

In a systematic review and network meta-analysis involving 5598 infants and 30 trials, various NIV strategies (nCPAP, INSURE, LISA, nIPPV, nebulized surfactant, surfactant administration via laryngeal mask) and IMV were evaluated. It was found that the use of LISA (+nCPAP) was associated with the lowest likelihood of the composite outcome of death or BPD at 36 weeks’ postmenstrual age (PMA). Moreover, the secondary outcomes of BPD and severe IVH were lower with LISA (+nCPAP) than with IMV, and there was a lower likelihood of death or BPD at 36 weeks and air leak with LISA (+nCPAP) than with nCPAP alone. Yet, these findings were limited by the overall low quality of evidence [124]. Another meta-analysis found that the application of the LISA technique in infants with RDS is associated with a decreased need for IMV and improved composite outcome of death or BPD at 36 weeks and survival without BPD [53]. In addition, a recent RCT in extremely preterm infants treated with either LISA or conventional endotracheal intubation assessed the psychomotor development at the age of 24 months. It was found that the LISA-treated infants scored higher on the psychomotor development index (PDI) and mental development index (MDI) [125]. With the recently published Cochrane review, in which the administration of surfactant via thin catheter compared with the administration via an endotracheal tube was found to significantly reduce the risk of death or BPD, intubation in the first 72 h of life, severe IVH, BPD in survivors as well as in-hospital mortality [126], and the pending results of the OPTIMIST-A trial, it is anticipated that the clinical use less invasive surfactant administration will be expanded.

#### 5.2.3. Use of Nebulized Surfactant

Another non-invasive method for surfactant administration is the administration of aerosolized surfactants. During the last decades, attempts to treat RDS with aerosolized surfactant using different nebulization methods failed to prove the undisputed effectiveness of this method. Only recently, randomized clinical studies using modern nebulization methods demonstrated the feasibility and effectiveness of aerosolized surfactant nebulization in preventing intubation and mechanical ventilation [54]. The study by Cummins et al. that enrolled 457 neonates with a wide range of GA (23 to 41 weeks, median 33 weeks) showed that in neonates with mild-moderate respiratory distress, aerosolized surfactant decreased the intubation rate and surfactant instillation by 50% [55]. There is also an ongoing systematic review and meta-analysis on surfactant nebulization that may further clarify the feasibility and effectiveness of nebulized surfactants for treating RDS [56].

## 6. The Role of the Adjunctive Pharmacological Interventions

Various medications (caffeine citrate, vitamin A, etc.) have been evaluated as to their ability to either improve survival or prevent complications associated with IMV, mainly BPD in preterm infants [127]. An in-depth reference into the full list of pharmacological treatments is beyond the scope of this article. However, inhaled nitric oxide (iNO), postnatal steroids, and oxygen should be mentioned, as they play an integral role in the respiratory care of sick preterm and term neonates (Table 1). 

### 6.1. Inhaled Nitric Oxide

The administration of iNO is an established therapy that has been proven effective at reducing the need for extracorporeal membrane oxygenation in infants born at or near term with hypoxic respiratory failure unresponsive to other therapies [128]. iNO has also been used as a rescue therapy in premature infants with persistent respiratory failure and for the prevention of BPD. Existing evidence, though, does not support the use of iNO in preterm infants for any of these other indications—especially for the prevention of BPD [57]. However, iNO could be used for the treatment of acute, severe pulmonary hypertensive crises in established BPD. Due to known limitations of providing iNO, including its high cost, alternative vasodilator therapies may be preferable [58].

### 6.2. Postnatal Steroids

Due to the central role inflammation plays in the development of BPD, postnatal steroids are given to at-risk infants to mitigate inflammation and decrease the likelihood of BPD. Historically, relatively high doses of dexamethasone were used as therapy in infants with BPD [129]. Various therapeutic protocols have since been tried, with significant differences regarding the type (dexamethasone, hydrocortisone), timing (early, late), dose (high, low), and route of administration (systemic, inhaled, with surfactant). 

Existing data show that early (prior to the day of life 8) systemic postnatal corticosteroids reduce the risk of BPD without affecting mortality. Dexamethasone, however, might significantly increase the risk of cerebral palsy [130]. A Cochrane review of late (>7 days) postnatal systemic corticosteroid treatment for BPD documented important benefits, including reduced extubation failure, BPD, and death or BPD, at both 28 days of life and 36 weeks’ PMA. Although no overall reduction in neonatal mortality has been achieved, the risk of adverse long-term neurodevelopmental outcomes was not found to have significantly increased, which is an extremely important issue [59]. A double-blind, placebo-controlled, randomized trial of early low-dose hydrocortisone in extremely preterm infants (PREMILOC) reported a significant increase in survival without BPD at 36 weeks of PMA. Of note, 80% and 82% of the infants in the hydrocortisone and placebo groups, respectively, were intubated before study entry. Moreover, there were no differences between groups regarding adverse events, including gastrointestinal perforation and sepsis, with the exception of a significantly higher rate of infection in the subgroup of treated infants born at 24 to 25 weeks [60]. In another double-blind RCT, where hydrocortisone therapy was initiated 7 to 14 days after birth in very preterm infants receiving IMV (STOP-BPD Study), specific treatment did not improve the composite outcome of death or BPD at 36 weeks’ PMA [131].

The effect of inhaled corticosteroids on BPD and mortality in ventilated VLBW infants, when given early during the first two weeks of life, was found to be comparable to that of systemic steroids, even though the duration of respiratory support and patent ductus arteriosus rate increased with inhaled steroids. Furthermore, in the long-term, inhaled steroids were associated with a lower rate of asthma and similar neurodevelopment at the age of 7 years [61]. However, there are concerns about an increase in mortality when inhaled budesonide is started within 24 h of birth for the prevention of BPD in ELBW infants [132]. Another method for steroid administration in preterm infants to decrease BPD is the intratracheal instillation via surfactant, which is currently under investigation by the PLUSS trial. This is a multicenter, double-blind, two-arm, parallel 1:1 placebo-controlled randomized trial conducted in Australia and New Zeeland to compare the safety and efficacy of early intratracheal corticosteroid instillation using exogenous surfactant as the vehicle vs. exogenous surfactant alone (Trial registration: ANZCTR: ACTRN12617000322336, available at https://www.anzctr.org.au, accessed on 10 September 2021) [62].

### 6.3. Oxygen Therapy: Saturation Targets and Automated Control

In recent years, scientific interest has focused on the relationship between the oxygen saturation of the blood (SpO₂) and the various outcomes of extreme prematurity, including BPD. Major concerns were raised after the reporting of higher mortality and an increased rate of NEC in infants born before 28 weeks’ gestation with targeted oxygen saturation below 90%. Interestingly, no difference in BPD was noted between the lower-target group (85% to 89%) and the higher [63]. A Cochrane meta-analysis also concluded that in extremely preterm infants, targeting lower SpO₂ levels increased the average risk of mortality while decreasing the incidence of ROP requiring treatment. However, there was no significant effect on the composite outcome of death and/or major disability or on major disability alone [36].

In this context, automated oxygen controllers have also been used as a means to increase the time spent within the SpO₂ target range, avoiding both hypoxemia and hyperoxemia and their associated increase in mortality and other prematurity-related morbidities. As a matter of fact, as documented in a recent randomized crossover trial in preterm infants, the utilization of automated oxygen controllers in ventilated infants resulted in significantly fewer desaturations lasting > 30 s and more time spent in the SpO_2_ target range. However, there was no significant effect on the quantity of blood gases, or the number of chest radiographs obtained [37]. In a retrospective cohort study in which clinical outcomes of preterm infants were evaluated before and after the implementation of automated oxygen control as the standard of care, invasive ventilation was significantly shorter in infants who received automated oxygen control. There was no difference, however, in the length of stay in the NICU, mortality, or morbidity during the study periods [64]. On the contrary, in another retrospective cohort study, improvement in rates of mortality, any ROP, ROP requiring treatment, and BPD was reported after implementation of a specific novel oxygen management strategy [65].

Overall, closed-loop automated oxygen control seems to improve target saturation in preterm infants on positive pressure ventilation and might substantially reduce nursing workloads [133]. Answers regarding the effect of closed-loop automated control of FiO_2_ on clinically important outcomes in the short- (NEC and BPD at 36 weeks PMA, severe ROP at the completion of retinal vascularization) and long-term (composite outcome of death, neurodevelopmental impairment at 24 months corrected age) will be provided by an ongoing multi-center RCT (Trial registration: www.ClinicalTrials.gov: NCT03168516) [134].

## 7. Current Role of Invasive Mechanical Ventilation

Unfortunately, in a significant proportion of preterm infants initially treated non-invasively, nCPAP will fail (especially in the sickest and more immature) [134]. nCPAP failure has been associated with a substantially higher rate of pneumothorax, death, BPD, and other morbidities, as well as longer durations of respiratory support and hospitalization, compared with successful nCPAP management [135]. FiO₂ ≥ 0.3 during the first 2 and 6 h was highly predictive of nCPAP failure for infants born at 25–28 and 29–32 weeks’ GA, respectively [136].

Possibly, the integration into clinical practice of novel tools, such as the lung ultrasound, may allow a more accurate and personalized management of RDS, thus reducing nCPAP failure and the need for IMV [137], leading to a decline in the overall mortality and deaths related to pulmonary causes among extremely premature infants. Interestingly, RDS and BPD were the second most common causes of death following immaturity [138], emphasizing the dominant role of IMV in the management of respiratory failure in preterm and term neonates.

## 8. Cost and Benefit of Neonatal Care-Respiratory Support

The cost factor is undoubtedly of particular importance both in periods of economic stability and instability, especially when it comes to intensive care and low-income countries. Preterm infants are among the most expensive pediatric patients [139,140]. Increased survival in premature infants is directly linked to longer hospital stays and, therefore, higher costs [13]. A desirable aspect of intensive care in neonates is that high costs seem to be rather “justified” due to the good outcome of most critically ill infants. As commented by Buchh et al. in a relevant article on resources, “*surprisingly, even when NICU survival was much worse, there have never been credible distributive justice arguments against NICU care for infants with BW <1000 g, whether dollars spent on survivors or ‘intact survivors’ is the outcome measure*” [141]. In any case, when evaluating the value of NICU care using quality-adjusted life years (QALY), neonatal intensive care for term infants has been calculated to $1000 per QALY and intensive care of extremely preterm infants to $9100, which is considered very cost-effective [142].

In terms of respiratory support, the application of potentially best practices, such as NIV, might reduce not only respiratory complications but also costs associated with the use of expensive drugs (e.g., iNO) [143] and respiratory equipment (ventilators versus simpler devices) [144]. A big retrospective study from the national Kid’s Inpatient Database of the Healthcare Cost and Utilization Project, for the years 1997–2012, showed that the application of only NIV was associated with shorter length of stay, decreased costs, and decreased charges compared to intubation and IMV [94]. Nevertheless, in another study evaluating different non-invasive respiratory support measures, the mean cost of hospitalization per infant was similar between nIPPV and nCPAP ($143,745 and $140,403, respectively). Interestingly, a cost-effectiveness evaluation revealed a 61% probability that nIPPV not only costs more but is less effective than nCPAP [145].

## 9. Future Challenges and Perspectives

The advances in the respiratory support of neonates, as a consequence of the progress made on the overall scientific understanding of neonatal physiology and medical technology, have significantly contributed to a better outcome for critically ill neonates. Nevertheless, not all respiratory practices are based on strong evidence supporting their application to the littlest of patients. 

The lack of generally accepted outcome definitions, such as the BPD definition, and the rarity of lung pathology documentation have been important drawbacks in neonatal respiratory research. Globally used definitions proposed by executive workshops can contribute to the design of more pragmatic studies on respiratory support and, ultimately, short-and long-term outcomes of vulnerable preterm infants.

New generation ventilators providing constant and objective monitoring of the respiratory system will possibly lead to better clinical decisions. Additionally, with the use of available demographic and clinical-laboratory data, learning machine tools may allow the development of predictive models of clinically important outcomes and recognize high-risk populations. Some promising relevant studies regarding extubation readiness in preterm infants have already been published [146,147]. As for BPD, most clinical models of the past were reported to be poor to moderate predictors [148]; but new, promising studies with simple-to-use validated nomograms for predicting BPD in the early stage have emerged [149,150]. The integration of new technologies into clinical practice is not something in the distant future but will very soon be part of routine neonatal care.

Lastly, something to look forward to in the future, which would completely revolutionize the way we support extremely premature infants, may be found with the recent development of an extra-uterine system that can physiologically support extremely premature animals (lambs), allowing for normal growth and development, including lung and brain maturation [151].

## 10. Conclusions

The advances in respiratory support of neonates have contributed to a dramatic increase in survival, especially of the very low birth weight infants. However, despite all the progress, BPD still continues to be a clinically significant complication of prematurity with important long-term consequences. Given that there are, and there will continue to be, high-risk pregnancies and a considerable number of infants will still require respiratory support, either invasively or non-invasively, the management should include all known protective lung strategies and other evidence-based adjunctive treatments. 

In the 60 years of neonatal respiratory support, the pathway may appear slow, and the benefits in terms of the various morbidities may not be so impressive. Nevertheless, there have been important scientific leaps forward. The general feeling is that there is room for greater improvements in the way infants with respiratory failure are treated so that they survive with minimal or no lung injury or other complications, which could largely affect their quality of life later in adulthood.

## Figures and Tables

**Table 1:** Summary of the respiratory support modes employed so far and their effect on neonatal outcome.

Mode	Effect on Outcome	Selected References
**Invasive Mechanical Ventilation (IMV) modes**		
Conventional IMV	Increased survival, ROP & neurodevelopmental impairment; unclear effect on BPD.	[1,4,11,12,22]
Synchronized IMV	Reduced air leaks & duration of IMV, unclear effect on BPD.	[25]
*NAVA*	None	[26,27,28]
*PAV*	Unknown	[29]
VTV (VCV & VG)	Reduced BPD, IMV duration, pneumothorax, death/BPD, brain damage.	[30,31,32]
HFV	Little effect on reducing lung damage (despite improvement of alveolar stability), better lung function at 11–14 years of age.	[33,34,35]
HFV + VG	Unknown	[36,37]
**Non-Invasi** **ve Ventilation (NIV) modes**		
nCPAP	Reduced BPD at 36 wks, death or BPD.	[38,39]
Bi-level NIV		
*nIPPV*	Reduced risk of respiratory failure and intubation. No effect on BPD.	[40,41]
*Bi-PAP*	Effects comparable to nCPAP.	[42]
nHFV	Reduced duration of NIV, need for IMV, incidence of IVH.	[43,44,45]
High flow nasal cannula (HFNC)	HFNC as primary respiratory support: Effect on mortality, BPD, similar to other NIV modes; longer respiratory support. HFNC post extubation: Reduced nose trauma and pneumothorax, longer treatment compared to nCPAP.	[46,47,48]
nNAVA	Similar to nCPAP	[49,50]
**Alternative exogenous surfactant administration modes**		
INSURE	Increased rate of extubation failure.	[51,52]
LISA / MIST (+ nCPAP)	Decreased mortality or BPD at 36 wks PMA, BPD, severe IVH	[53]
Nebulized surfactant	Decreased intubation rate by 50% in mild-moderate respiratory distress. Ongoing metanalysis..	[54,55,56]
	**Adjunctive treatments**	
iNO	Evidence does not support the use of iNO for BPD prevention. iNO could be used for severe PPHN in established BPD.	[57,58]
Postnatal steroids		
*Systemic*	Early (< 4 DoL): Reduced BPD, no effect on mortality, possibly increased risk of cerebral palsy. Late (>7 DoL): Reduced extubation failure, BPD, and death or BPD, without adverse long-term neurodevelopmental outcomes.	[59,60]
*Inhaled*	Early (1–14 DoL): Compared to systemic steroids, similar effect on BPD, longer respiratory support, increased PDA rate; lower rate of asthma & similar neurodevelopment at 7 years of age.	[61]
*Endotracheally*	Endotracheal installation via surfactant is currently under investigation (PLUSS trial).	[62]
Oxygen		
*Low vs. high target*	No difference in BPD. Low SpO_2_ (SpO_2_ 85–89%): Increased mortality, decreased ROP.	[36,63]
*Automated oxygen control*	Contradictory results: Either no effect or decreased mortality, any ROP, ROP requiring treatment, and BPD.	[37,64,65]

BPD, Broncho Pulmonary Dysplasia; CPAP, Continuous Positive Airway Pressure; DoL, Day of Life; HFNC, High Flow Nasal Cannula; HFV, High-Frequency Ventilation; IMV, Invasive Mechanical Ventilation; iNO, inhaled nitric oxide; INSURE, Intubate–SURfactant–Extubate; IVH, Intra-Ventricular hemorrhage; LISA, Less Invasive Surfactant Administration; MIST, Minimal Invasive Surfactant Technique; n, nasal; NAVA, Neurally Adjusted Ventilatory Assist; nIPPV, nasal Intermitted Positive Pressure Ventilation; NIV, Non-Invasive Ventilation; PAV, Proportional Assist Ventilation; PMA, Post-Menstrual Age; ROP, Retinopathy Of Prematurity; VCV, Volume-Controlled Ventilation; VG, Volume Guarantee; VTV, Volume-Targeted ventilation.
